# Design and site-directed compartmentalization of gold nanoclusters within the intrasubunit interfaces of ferritin nanocage

**DOI:** 10.1186/s12951-019-0512-0

**Published:** 2019-07-05

**Authors:** Jiachen Zang, Bowen Zheng, Xiuqing Zhang, Paolo Arosio, Guanghua Zhao

**Affiliations:** 1 Beijing Advanced Innovation Center for Food Nutrition and Human Health, College of Food Science & Nutritional Engineering, Beijing Key Laboratory of Functional Food from Plant Resources, China Agricultural University, Beijing, 100083 China; 2 Department of Molecular and Translational Medicine, University of Brescia, Viale Europa 11, 25123 Brescia, Italy

**Keywords:** Compartmentalization, Intrasubunit interface, Interface redesign, Gold nanoclusters, Mitochondrial ferritin

## Abstract

**Background:**

Protein nanocages have emerged as popular nanocarriers for either drug delivery or biotemplates for the preparation of nanomaterials. However, only three interfaces, namely exterior surface, intersubunit and inner cavity, have been used as reaction sites for the above purposes with all known protein nanocages. On the other hand, how to control the site of Au NCs formed within a targeted protein template while maintaining the functionality of protein itself remains challenging.

**Results:**

In this work, inspired by compartmentalization in living systems, we firstly come up with the conception of “intrasubunit interfaces”, located within subunit of protein nanocage. We built a new, specific compartment for fabrication of gold nanoclusters by genetic modification of the inherent ferroxidase center located within four-α-helix bundle of each ferritin subunit. This newly built compartment not only realizes the site-directed synthesis of gold nanoclusters but also has no effect on the functionality of ferritin itself such as encapsulation by its inner cavity. These redesigned composites can be further applied as fluorescent imaging agent and carriers for preparation of hybrid nanomaterials.

**Conclusions:**

The designing strategy of intrasubunit interfaces opens a new way for future applications of cage-like proteins.

**Electronic supplementary material:**

The online version of this article (10.1186/s12951-019-0512-0) contains supplementary material, which is available to authorized users.

## Background

**Fig. 1:**
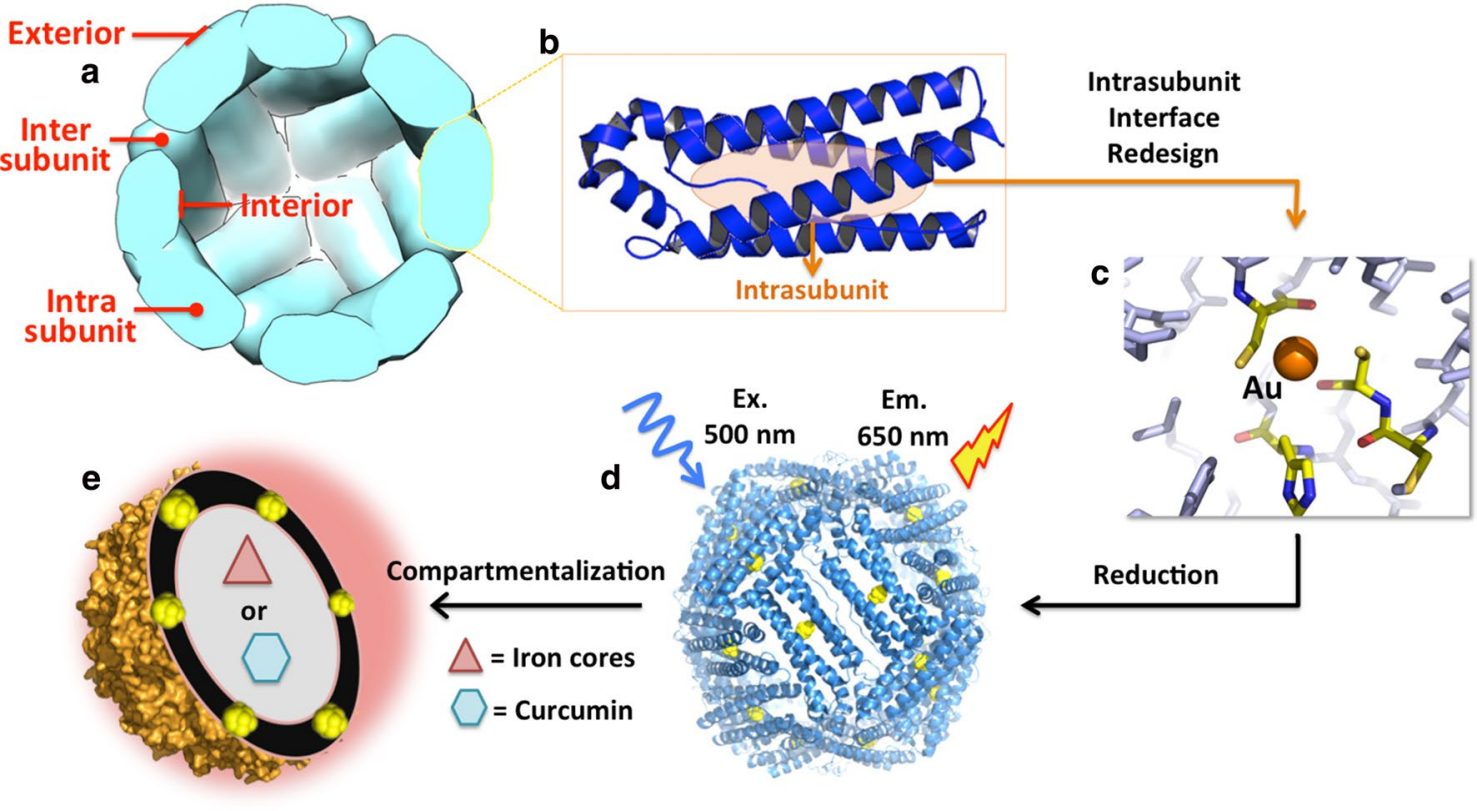
**a** Structure of ferritin verified by four reacting interfaces, in which the exterior, intersubunit and interior interfaces were reported to be used in a variety of applications. But the intrasubunit interface was put forward for the first time. **b** Highlight of intrasubunit interface within one subunit. **c** The ferroxidase center buried within the intrasubunit was redesigned into a new compartment which can specifically bind to gold ions by introducing Cys residues. **d** Site-directed synthesis of gold nanoclusters (Au NCs) within 24 four α-helix bundles of one ferritin molecule. **e** Compartmentalization of two different kinds of nanoparticles within one ferritin nanocage. The Au NCs were located within protein shell, while inorganic iron cores or bioactive organic cargoes were encapsulated within the ferritin inner cavity

Compartmentalization is a hallmark of living systems, which enables them not only to establish physical boundaries for the spatial separation of incompatible or opposing reagents to avoid mutual destruction, but also to perform the chemical reactions between these reagents simultaneously [1, 2]. Protein nanocages, a class of naturally occurring compartments, shield their cargo from the influence of external conditions and provide a controlled microenvironment. These protein nanocages are widely distributed in nature to fulfill a variety of functions [3–8]. Recently, the protein nanocages have received considerable attention from researchers in the field of nanoscience and nanotechnology due to their valuable properties such as high symmetry, solubility and stability, monodispersity, and ease of genetic and chemical manipulation. Therefore, these protein nanocages have been explored as biotemplates for the preparation of inorganic and organic nanomaterials, and the encapsulation and delivery of guest molecules with various potential applications [3, 4]. Although the protein nancages are markedly different from each other in size, assembly, and function, there is one structural feature in common among all reported protein nanocages, namely all of them have three distinct interfaces: the interior, the exterior and the intersubunit interface, as previously reported [3]. However, in this paper, to further develop the application efficiency of protein nanocage, namely build a new compartment within the cage shell for other nanomaterials, we come up with the fourth interface as “intrasubunit interfaces” at the first time (Fig. 1a).

Among these protein nanocages, the ferritin nanocage is unique in that it exhibits high selectivity for human cancer cells which overexpress two kinds of receptors for ferritin [9, 10]. Thus, it has emerged as a class of drug delivery vehicles and imaging agents. Ferritin ubiquitously occurs in animals, plants and bacteria, and plays a significant role in iron storage and detoxification [11]. It is a nearly spherical 24-subunits-protein with exterior diameter about 12 nm and a hollow cavity of 8 nm [12]. Owing to its cage-like morphology and highly symmetrical structure, ferritin displays outstanding thermal and chemical stability and it is remarkably amenable to pH induced dis- and re-assembly [13, 14]. Like other protein nanocages, ferritins also have four chemically distinct interfaces where can be manipulated in order to impart functionality by design (Fig. 1a). The intersubunit interfaces in ferritin consist of *C*_2_, *C*_3_, *C*_4_, and *C*_3_–*C*_4_ as shown in Additional file 1: Figure S1. Among these intersubunit interfaces, *C*_3_ and *C*_4_ are mainly responsible for diffusion of small molecules into ferritin cavity [15, 16], while *C*_2_ plays a key role in protein shell assembly [17]. The *C*_3_*–C*_4_ is the most largest intersubunit interface in ferritin, which controls protein geometry based on our recent studies [18, 19]. To extend the utilization of ferritin nanocage, protein exterior surface has been frequently modified so that it can be endowed with new functionalities such as specific targeting ability or luminescence [20–22]. As popular as the exterior surface, the inner cavity of ferritin has been explored to encapsulate various cargoes such as simple metal ions and complicated drugs [23–25]. However, so far, there have been no reports focusing on the intrasubunit interface of protein nanocages including ferritin prior to this study.

On the other hand, gold nanoclusters (Au NCs) are being actively pursued as a novel kind of fluorescent materials due to their potentially low toxicity, well-defined compositions, bright luminescence and facile surface functionalization [26–28]. After bovine serum albumin (BSA) was first exploited to be embedded with Au NCs in 2009 [29], substantial proteins have been used as biotemplates for Au NCs synthesis [30–33]. However, how to control the site and size of Au NCs formed within a targeted protein template while maintaining the functionality of protein itself remains challenging. Herein, we take the compartmentalization concept to re-design the intrasubunit interface of ferritin to create a new compartment for fabrication of fluorescent Au NCs, while keeping the inherent protein cavity functional. Consequently, we can incorporate binary nanomaterials into one ferritin nanocage, namely small Au NCs buried within the intrasubunit and large inorganic or organic nanoparticles (4–6 nm) encapsulated within the cavity, which provide a new way for construction of hybrid nanomaterails.

## Materials and methods

### Protein preparation

cDNA encoding the amino acid sequence of mature HuFtMt was cloned into the pET-3a (Novagen) and verified by DNA sequencing. Mutagenesis of the HuFtMt cDNA was performed with the fast site-directed mutagenesis kit from Tiangen Biotech (Beijing, China). The mutated HuFtMt and wild-type HuFtMt were purified as follows. The *E. coli* strain BL21(DE3) which contained the expression plasmid was grown at 37 °C. Protein expression was likewise induced with 200 μM of isopropyl β-d-1-thiogalactopyranoside after the cell density reached an absorbance of 0.6 at 600 nm. Then the cells were harvested by centrifugation after 8 h of induction and resuspended in 25 mM Tris–HCl (pH 8.0) to a concentration of 10 g fresh weight bacteria per liter, followed by disruption by sonication. The supernatant of the resulting crude extract was collected by centrifugation and fractionated by 60% saturation of ammonium sulfate. The pellet was resuspended in 25 mM Tris–HCl (pH 8.0) and dialyzed against the same buffer. The protein solution was applied to an ion-exchange column, followed by gradient elution with 0–0.3 M NaCl. Finally, the protein solution was concentrated and purified on a gel filtration column, equilibrated with 25 mM Tris–HCl and 150 mM NaCl (pH 8.0). Protein concentrations were determined according to the Lowry method with bovine serum albumin as standard.

### Synthesis of ferritin-stablized Au NCs

Ferritin stablized Au NCs were synthesized as follows. 200 μL of 10 mM aqueous HAuCl_4_ solution (pH value tuned to 7.0) was added to a 200 μL Tris–HCl solution containing protein (50 mg/mL) with vigorous stirring at 37 °C. 5 min later, pH value was adjusted to around 12.8 with further vigorous stirring for 20 min. After that, the mixture solution was incubated at 37 °C overnight. To increase the storage stability while terminating the reaction, 1.0 M acetic acid was added drop-wise until the pH value reached ~ 7.0. Resulting products were stored in dark at 4 °C for further use.

### Characterization of ferritin mutants and ferritin-stabilized Au NCs

UV–vis absorption spectra was recorded by a UV Spectrophotometer (Virian, 50 Bio, USA), and fluorescence studies in a sealed cuvette were carried out with a Fluorescence Spectrometer instrument (Virian, Palo Alto, USA). Fluorescence images were obtained under UV-light. Transmission electron microscope (TEM) images were obtained at 80 kV using a Hitachi S-5500 microscope. High-resolution transmission electron microscope (HRTEM) images were made by a JEM 2100F microscope, and elementary analysis was carried out with Horiba INCA 450 energy dispersive X-ray analysis spectroscopy. The oxidation state of the Au clusters was determined by X-ray Photoelectron Spectroscopy (XPS). Narrow-scan XPS spectra of Au 4f were deconvoluted by the XPSPEAK software (Version 4.1) using adventitious carbon to calibrate the binding energy of C1s (284.5 eV).

### Crystallization, data collection and structure determination

Purified proteins were concentrated to 10 mg/mL in a buffer consisting of 10 mM Tris–HCl at pH 7.6 and 1 mM HAuCl_4_. Crystals of wild-type HuFtMt and ΔC complexed with Au^3+^ were obtained using the hanging drop vapor diffusion method by mixing equal volumes of the complex sample and mother liquid, which was composed of 0.1 M Bicine-NaOH (pH 8.5) and 2 M MgCl_2_. Crystals of 2Cys-ΔC and 3Cys-ΔC complexed with Au^3+^ were obtained using the hanging drop vapor diffusion method by mixing equal volumes of the complex sample and mother liquid, which was composed of 0.1 M Tris–HCl (pH 8.5) and 3.4 M 1,6-hexalene glycol. Diffraction data of the crystal were collected at SSRF to resolutions of after flash cooling with 25% glycerol as a cryo-protectant. Data were processed, merged and scaled with the HKL-3000 (HKL Research). Data processing statistics are shown in Additional file 1: Table S1. The data was determined by molecular replacement using coordinates of wild-type Mfn (PDB code 1R03) as an initial model using the MOLREP program in the CCP4 program package. Structural refinement was conducted using the Refmac 5 program and PHENIX software. The structure was rebuilt using COOT, which made the model manually adjusted. Figures of protein structures were prepared using the PyMOL program.

### In vitro and in vivo bioimaging studies

HepG2 cells were cultured in DMEM supplemented with 10% (vol/vol) FBS, 2 mM l-glutamine and 1% penicillin/streptomycin and incubated at 37 °C with 5% CO_2_. Briefly, cells were seeded at a density of 3 × 10^5^ on the cell culture dish and pre-incubated for 24 h. Then the cells were treated with RGD decorated 3Cys-ΔC and 3Cys-ΔC-Au NCs for 3 h at 37 °C with 5% CO_2_ and then washed by PBS three times. After that, cells were fixed by 4% paraformaldehyde for 20 min at room temperature. Last, The confocal laser scanning microscopy was used to image the cells.

Wild-type *C. elegans* were cultured in the nematode growth medium (NGM) agar plates containing *E. coli* OP50 bacteria (OP50 medium) as food supplement at 20 °C. L4 stage worms were seeded in the NGM agar plates supplemented with 3Cys-ΔC as negative group and with 3Cys-ΔC-Au NCs as experimental groups. After cultivation for different times, worms were transferred onto microslide smeared with circled albolene and centered 20 mM NaN_3_ solution to fix the worms and then observed by laser-scanning confocal microscopy. The images of the 3Cys-ΔC-stabilized Au NCs treated sample and negative control samples were collected in the range of 575–620 nm with 488 nm as excitation wavelength.

### Iron cores formed within 3Cys-ΔC-stabilized Au NCs

Iron cores within 3Cys-ΔC-stabilized Au NCs were prepared by aerobically adding 500 Fe(II) per protein shell to 3Cys-ΔC-Au NCs solutions in five successive increments of 100 Fe(II) at intervals of 30 min in 50 mM MOPS at pH 7.0, followed by standing overnight. After iron loading, the sample was incubated at 4 °C in dark overnight because the iron mineralization was slower with ferritin mutants where the structure of ferroxidase center had been greatly altered.

### Preparation of curcumin-loaded 3Cys-ΔC-Au NCs

Curcumin-loaded 3Cys-ΔC-Au NCs were prepared as previously reported with curcumin encapsulation by human H-chain ferritin. Briefly, for a 1 mL system of alkaline as-prepared 3Cys-ΔC-Au NCs (1 mg/mL in 0.15 M NaCl), a 200 μL solution of curcumin, freshly solubilized in 0.1 M sodium hydroxide (2 mM), was added. Immediately, the pH value was lowered to 7.5 using 1 M HCl. Resulting solution was stirred at room temperature for 2 h to promote the assembly of the protein. Finally resultant products were run through a NAP-5 column to remove free curcumin.

### Thermal stability and photostability measurements

Both of curcumin encapsulated 3Cys-ΔC-Au NCs and free curcumin were heated at 60 °C in a dark tube. The degradation kinetic of curcumin was carried out by scanning UV–vis spectra from 250 to 800 nm at interval times. In the photostability experiment, the sample for photo degradation studies was placed under an incandescent lamp of 18 W/YH, at a distance of 10 cm at 25 °C. the degradation kinetic of curcumin was carried out by also scanning UV–vis spectra from 250 to 800 nm at interval times. As curcumin is poorly soluble in water, a solution of curcumin dissolved in ethanol at 10 mM was used as stock solution and then diluted to same intensity at ~ 425 nm with curcumin encapsulated 3Cys-ΔC-Au NCs to act as a control sample.

## Results and discussion

### Design of ferritin-templated Au NCs

In this work, we selected the relatively rare reported human mitochondrial ferritin (HuFtMt) nanocage as the protein model based on the following considerations: (1) it is a naturally occurring homopolymer which can simplify the design challenge; (2) its sequence and crystal structural information are available to guide genetic manipulations; and (3) it could be a promising vehicle for tumor imaging and drug delivery due to its nonimmunogenicity. Of particular interest is the site-directed synthesis method of Au NCs (Fig. 1). This involves genetically modifying the ferroxidase center buried within the intrasubunit interface into a compartment which is suitable for growth of Au NCs. To our knowledge this method has never previously been used to ‘trap’ any NCs and could be implemented to fabricate NCs within the intrasubunit interface of multisubunit protein architectures. More importantly, we demonstrate that incorporation of Au NCs into the intrasubunit interface imparts highly luminescent property to ferritin while having no effect on both the formation of inorganic nanomaterials (iron cores) and encapsulation of bioactive molecules (curcumin) within the cavity (Fig. 1e).

Au NCs are typically less than 2 nm in size which approaches the Fermi wavelength of the conduction electrons. Although the cavity of ferritin serves as an ideal nanocontainer for synthesis of various nanoparticles [3, 4], it is not suitable for preparation of Au NCs because the size of the cavity is around 8 nm in diameter which is too large for such preparation. On the other hand, once the cavity is occupied by Au NCs, the ability of ferritin to encapsulate guest molecules will be greatly compromised. Similarly, it is also difficult to control the size of Au NCs on the exterior surface of ferritin. Although the pore size of various ferritin channels (3- or 4-fold channels) located at the intersubunit interfaces (Additional file 1: Figure S1) is similar to that of Au NCs, occupation of these channels by Au NCs will inhibit diffusion of metal ions into the cavity for mineralization. By investigating all interfaces of HuFtMt, we found that the ferroxidase center buried within the intrasubunit interface appears to meet all kinds of requirements for fabrication of Au NCs. Firstly, the ferroxidase center is located in the middle of four α-helix bundle of each subunit, which is some 7 Å from the inside surface of the protein shell and ~ 12 Å from the exterior surface in a region of considerable hydrophobic character, providing a size-constrained reaction platform for Au NCs synthesis. Secondly, when Au NCs were generated at the ferroxidase center, they would not affect the utilization of the ferritin cavity.

**Fig. 2:**
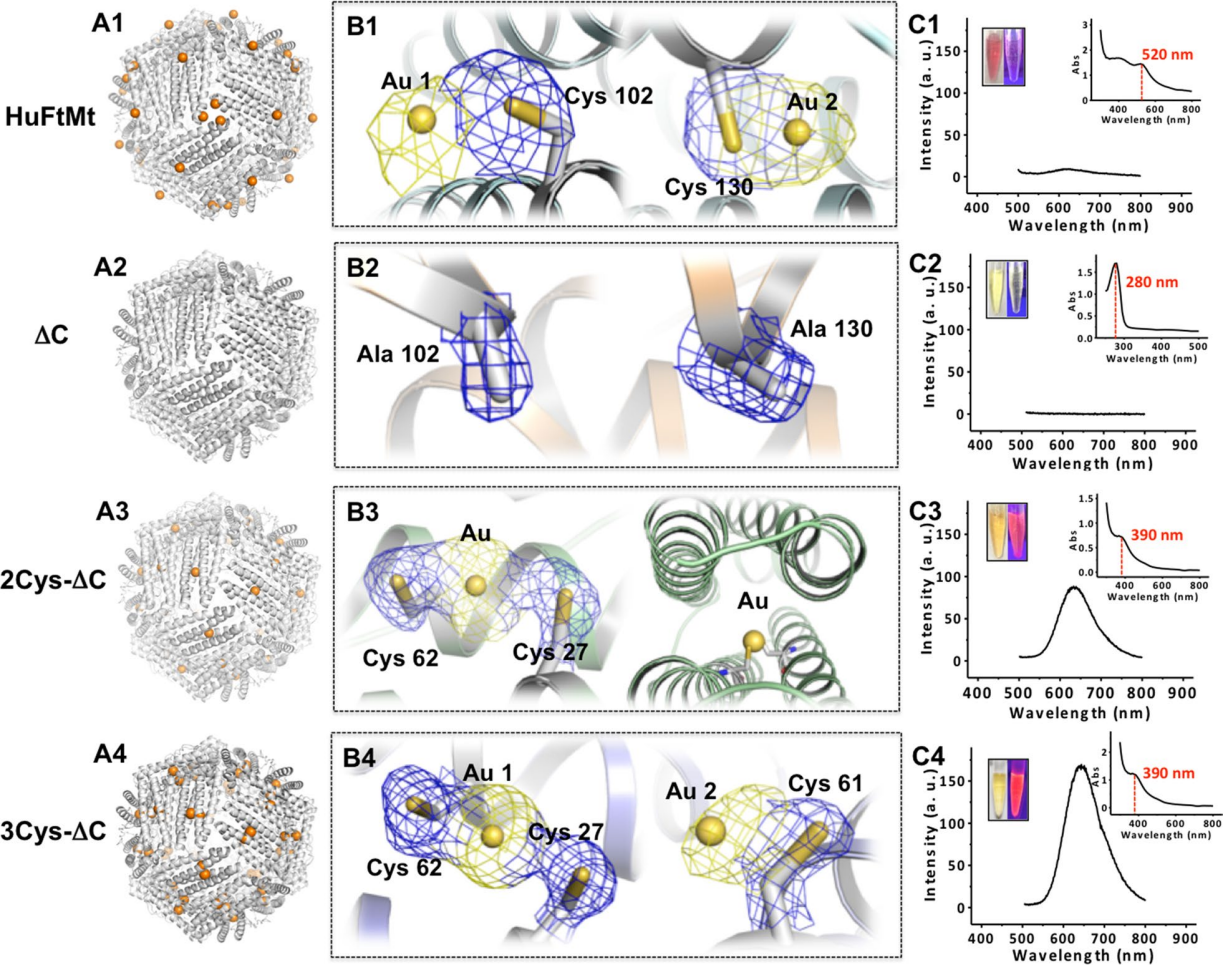
Crystal structure of HuFtMt (**A1**), ΔC (**A2**), 2Cys-ΔC (**A3**), and 3Cys-ΔC (**A4**) treated with HAuCl_4_, respectively. Au ions are highlighted in orange. **B1** Highlighting two gold ion (yellow) coordination sites where Cys102 and Cys130 are involved. **B2** No Au ion binding was observed at the location of Ala102 and Ala130 in mutant ΔC. **B3** Highlighting one Au ion (yellow) binding site at the modified ferroxidase center of 2Cys-ΔC from two different views (side-view, left; top-view, right). **B4** Highlighting two Au ion (yellow) binding sites and involved amino acid residues at the modified ferroxidase center of 3Cys-ΔC. Fluorescent and UV–vis absorption spectra of HuFtMt (**C1**), ΔC (**C2**), 2Cys-ΔC (**C3**), and 3Cys-ΔC (**C4**) treated with HAuCl_4_ at basic condition, respectively. Inset shows two photo of the mixture under daylight (left) and UV-light (right)

Like human H-chain ferritin, the ferroxidase centers of HuFtMt are composed of A and B iron binding sites of conserved amino acid ligands His65, Glu27, Glu107, Glu61 and Glu62 (Additional file 1: Figure S2) [34, 35]. H-bonding residues Gln141 and Tyr34 are nearby. Besides iron ions, other metals such as magnesium, calcium and zinc can also bind at these centers [36]. To determine whether wild-type (wt) HuFtMt are able to bind to Au ions, we solved the crystal structure of wt HuFtMt at a resolution of 3.0 Å upon protein treatment with gold ions. The refined structure revealed two Au ions binding sites where Cys102 and Cys130 are involved in this protein as shown in Fig. 2A1 and B1, demonstrating that Au ions exhibits the high binding selectivity for cysteine residues. Therefore, to create specific Au ions binding site at the ferroxidase center, we re-designed these centers by genetically introducing cysteine residues as potential coordination ligands into them under help of the YASARA software in two steps. First, to eliminate possible non-specific binding of Au ions, we made a recombinant HuFtMt mutant termed ΔC (C102A/C130A) as a starting plat-form, which is devoid of any cysteine residues. Second, given the binding complexity of gold ions, we prepared a number of the ferroxidase center mutants. We found that both substitution mutants 2Cys-ΔC (C102A/C130A/E27C/E61C) and 3Cys-ΔC (C102A/C130A/E27C/E61C/E62C) are soluble; in contrast, the majority of two 4Cys-ΔC mutants (C102A/C130A/E27C/E61C/E62C/H65C and C102A/C130A/E27C/E61C/E62C/Q107C) remains in inclusion body pellets even when 0.5 M sorbitol were included in the growth medium, so we focus on the first two mutants.

Genes encoding the designed proteins (ΔC, 2Cys-ΔC, and 3Cys-ΔC) were constructed and cloned into an expression vector, *Escherichia coli* (*E. coli*) BL21 cells, respectively. After *E. coli* cells expressing the proteins were lysed, the proteins were purified by a combination of gel and ion-exchange chromatography. As expected, SDS-PAGE analyses revealed that all these three mutants consist of one type of subunit (Additional file 1: Figure S3A). Native PAGE of these proteins exhibited a single band, indicating that they were purified to homogeneity (Additional file 1: Figure S3B). As designed, these mutants are indistinguishable from the wt protein in terms of assembly, structure, and morphology based on fluorescence and transmission electron microscopy (TEM) analyses (Additional file 1: Figure S3C and S3D).

It is believed that a small nanocluster in protein scaffolds are formed by binding of metal ions, followed by nucleation [37, 38]. Therefore, we firstly determined whether Au ions can specifically bind at the modified ferroxidase center. To this end, we obtained the crystals through co-crystallization of three mutants ΔC, 2Cys-ΔC and 3Cys-ΔC with Au^3+^, respectively. ΔC shared a similar method with wt HuFtMt. However, for 2Cys-ΔC and 3Cys-ΔC, initial co-crystallization trials gave only small crystals, but large single crystals were eventually obtained, which are suitable for X-ray diffraction studies. Subsequently, we solved the crystal structure of these three samples at a resolution of higher than 2.3 Å. As expected, no Au ions binding was observed with ΔC mutant (Fig. 2A2 and B2) because the inherent binding residues Cys102 and Cys130 in wt ferritin had been replaced by Ala. Differently, the crystal structure of 2Cys-ΔC shows that one gold atom is coordinated by Cys27 and Cys62 in a linear geometry, occupying the original A site of the ferroxidase site, and this Au ion was buried within four α-helix bundle of each subunit (Fig. 2B3). There are totally 24 Au ions occurring at each protein shell as shown in Fig. 2A3. In contrast, two gold atoms are bound at the modified ferroxidase center of each 3Cys-ΔC subunit. One atom, named Au1, was located at the site A of the ferroxidase center, which is coordinated by Cys27 and Cys62 also in a linear geometry, while another gold atom, Au2, interacted with Cys61, nearby site B (Fig. 2B4). Consequently, each 3Cys-ΔC molecule can accommodate up to 48 Au ions (Fig. 2A4). Thus, these crystal structures validated our re-design of the ferroxidase center in atomic detail, thereby supporting the utility of the intrasubunit interface strategy for creating the specific Au binding sites.

Subsequently, we determined the ability of these three mutant proteins and wt HuFtMt as biotemplates to synthesize Au NCs, respectively, following our reported method by mixing protein and HAuCl_4_ at basic conditions and a further incubation at 37 °C overnight [32]. Consistent with the above crystal structure results, no fluorescence was observed with ΔC due to lack of Au ions binding in protein molecule (Fig. 2C2). In contrast, the 2Cys-ΔC-templated Au NCs emit a weak fluorescence under the same experimental condition (Fig. 2C3), which has an UV–vis absorbance at 390 nm, which is the characteristic surface plasmon resonance peak of small metal NCs (Fig. 2C3, inset). Among these three mutants, the as-prepared 3Cys-ΔC-stabilized Au NCs emit the strongest red fluorescence at 650 nm (Fig. 2C4). Thus the crystal structure ensure the Au NCs were synthesized within the subunit, namely intrasubunit.

### Characterization of 3Cys-ΔC-stabilized Au NCs

**Fig. 3:**
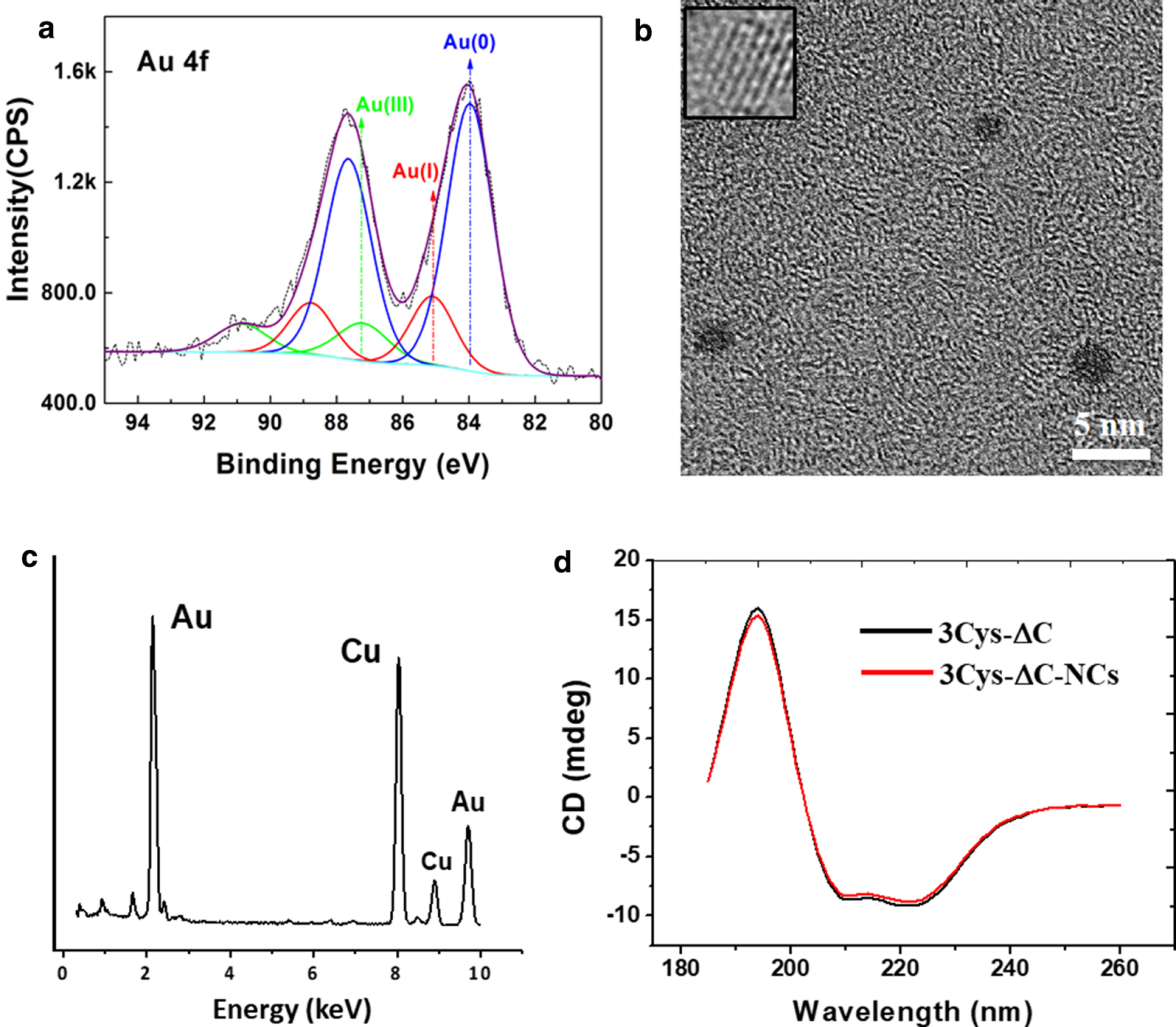
**a** XPS spectrum of 3Cys-ΔC-templated Au NCs. **b** HTEM image of 3Cys-ΔC-templated Au NCs. Inset shows lattice fringe of this newly synthesized ferritin-Au NCs composite. **c** EDX spectrum of 3Cys-ΔC-templated Au NCs. **d** Comparison of CD spectrum of 3Cys-ΔC-templated Au NCs with that of wt HuFtMt

Due to its strongest fluorescence among these three samples, we characterized 3Cys-ΔC-stabilized Au NCs with different physico-chemical methods. As shown in Additional file 1: Figure S4, to obtain the strongest emission peak at 650 nm, the maximum excitation wavelength was detected at 500 nm, which is beneficial for its application as an in vivo imaging agent. To illuminate the gold state in the hybrid, we run XPS spectrum with this new type of Au NCs. The 4f_7/2_ and 4f_5/2_ binding energies were examined, in which, Au(0) accounts for more than 70%, marked in blue (Fig. 3a). The Au(I) in red line ranked the second, confirming most of the Au(III) was reduced to be Au(I) and Au(0) during Au NCs synthesis. The TEM view of 3Cys-ΔC-stabilized Au NCs showed that Au NCs were monodispersed under basic conditions, while they were associated with each other once pH was adjusted to neutral (Additional file 1: Figure S5). To characterize the core size, the as-synthesized Au NCs were analyzed by high resolution transmission electron microscopy (HRTEM). According to Fig. 3b, the 3Cys-ΔC-stabilized Au NCs had spherical shape and were homogeneous in size, which was defined in the range of NCs (< 2 nm). Lattice fringes can be clearly visualized in HRTEM images (Fig. 3b, inset), indicating the formation of nanocrystalline materials. Energy-dispersive X-ray (EDX) analysis confirmed the presence of gold (Fig. 3c). The CD spectrum of the 3Cys-ΔC-templated Au NCs was nearly overlapped with that of protein template alone (Fig. 3d), indicating that protein secondary structure remained almost unchanged upon incorporation of small Au NCs into the intrasubunit. Therefore, the genetically modified ferroxidase center containing cysteine residues could be developed into a compartment to synthesize Au NCs. Based on these results, it is worth noting that Au ions preferentially bind to cysteine residues in protein, and such property can be utilized for directing the site of Au NCs formed with protein as a biotemplate.

**Fig. 4:**
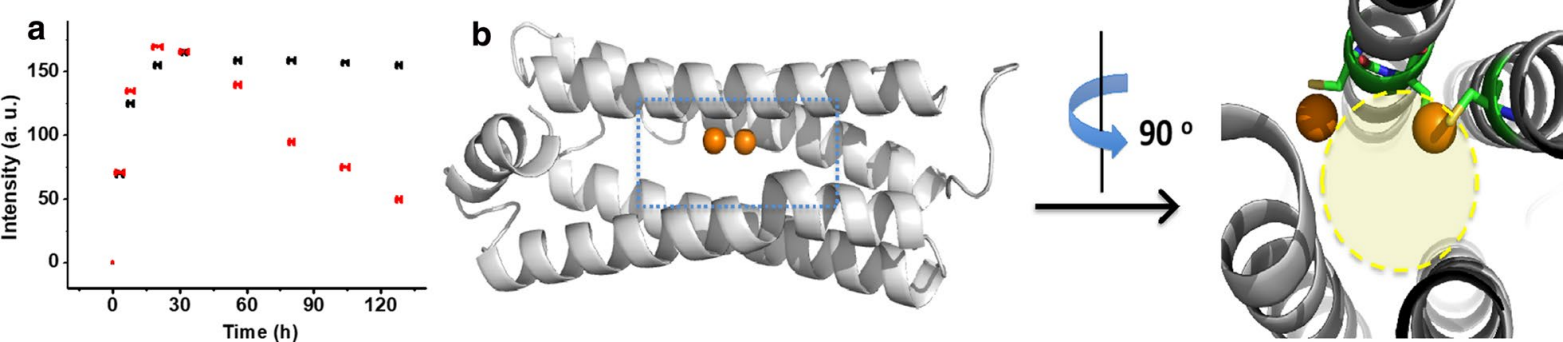
**a** The fluorescence intensity of 3Cys-ΔC-templated Au NCs (black) and β-lactoglobulin-templated Au NCs (red) as a function of reacting time under basic conditions. **b** The two Au binding sites are located within four α-helix bundle of 3Cys-ΔC observed from side-view and top-view, respectively, and such a size-constrained microenvironment is necessary for preparation of Au NCs

Compared with previously reported protein-templated NCs, 3Cys-ΔC-stabilized Au NCs is endowed with an outstanding feature as shown in Fig. 4a; namely, with continuous reduction by excess NaOH at 37 °C, the fluorescence intensity remains stable. However, for reported BSA or β-lactoglobulin their solution pH must be tuned back to neutral on time to terminate the reduction process, otherwise the formed NCs will grow into larger nanoparticles, leading to loss of fluorescence [29, 32]. Indeed, the fluorescence of β-lactoglobulin stablized Au NCs usually exhibits a different kinetic curve from 3Cys-ΔC-stabilized Au NCs as shown in Fig. 4a, namely, it initially increases to a maximum value at about 20 h, followed by decay. We believe that the higher stability of the 3Cys-ΔC-templated Au NCs stems from the prevention of the excessive growth of Au NCs by the size-constrained ferroxidase centers which are buried within four α-helix bundle (Fig. 4b). Consistent with this view, it was observed that the fluorescence of 3Cys-ΔC-stabilized Au NCs sharply decreased at 8 h (Additional file 1: Figure S6) upon increasing solution pH to 13.5, a value which can denature four-α-helix bundle. Further support for this view comes from fluorescence results when wt HuFtMt was used as a biotemplate for preparation of Au NCs. The mixture of wt HuFtMt with HAuCl_4_ exhibited a red color, with extremely weak fluorescence under UV-light as confirmed by its fluorescence spectrum (Fig. 2C1). This is most likely derived from the formation of larger-sized Au particles in the presence of wt HuFtMt. Indeed, the UV–vis spectrum of the mixture of wt HuFtMt with Au ions exhibits a peak at ~ 520 nm (Fig. 2C1, inset), which is characteristic of the larger-sized Au particles rather than NCs [39, 40]. This result is not surprising. As shown in Additional file 1: Figure S7A, three Au ions-coordinated Cys130 residues are located at the ferritin threefold channel (a relatively unconstrained microenvironment) [41], facilitating the excessive growth of Au NCs (Additional file 1: Figure S7B), thereby losing luminescent property. These findings emphasize the crucial role of the size-constrained domain such as the ferroxidase center buried within the intrasubunit in Au NCs synthesis.

### Imaging ability of 3Cys-ΔC-stabilized Au NCs

**Fig. 5:**
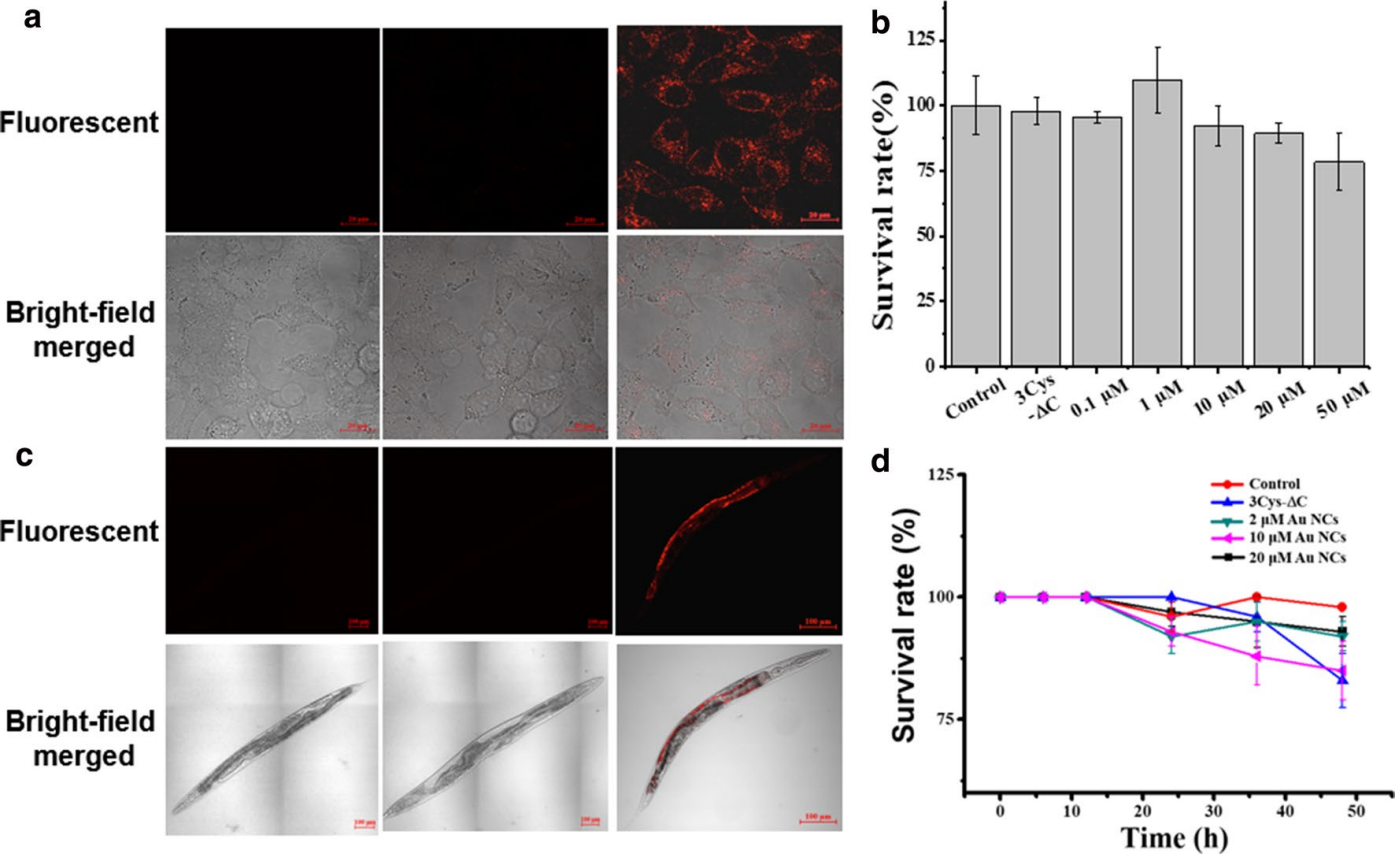
**a** Images of buffer as control, 3Cys-ΔC and 3Cys-ΔC-Au NCs treated HepG2 cells. Fluorescent and bright-field merged images were made by confocal microscopy. **b** Survival rate of cells treated with control buffer, 3Cys-ΔC and 3Cys-ΔC-Au NCs at differential concentrations. **c** Images of *E. coli* alone, complex of 3Cys-ΔC and *E. coli,* and complex of 3Cys-ΔC-Au NCs and *E. coli* in *C. elegans*. Fluorescent and bright-field merged images were made by confocal microscopy. **d** Survival rate of worms treated with control *E. coli*, 3Cys-ΔC and 3Cys-ΔC-Au NCs at differential concentrations

To increase the storage stability of 3Cys-ΔC-stabilized Au NCs while terminating the reducing reaction of HAuCl_4_ at basic conditions, the solution pH was adjusted back to ~ 7.0 by drop addition of 1 M acetic acid according to our reported method [32]. After negatively stained by uranyl acetate, the intact protein cage of 3Cys-ΔC-stabilized Au NCs was clearly visible as a white circles (Additional file 1: Figure S8A), indicating that ferritin nanocage is highly robust and can withstand the basic conditions used for preparation of Au NCs. Having thus incorporated Au NCs into the intrasubunit interfaces without perturbing the ferritin shell-like structure, we sought to identify the ability of 3Cys-ΔC-Au NCs to act as an imaging agent in vitro and in vivo. First, we incubated 3Cys-ΔC and 3Cys-ΔC-stabilized Au NCs with HepG2 cells, respectively. PBS buffer treated cells were set as control. As shown in Fig. 5a, even three samples displayed similar cell morphology in bright field, only 3Cys-ΔC-stabilized Au NCs incubated cells showed luminescence within the cytoplasm, which proved the fluorescence stability of ferritin stabilized Au NCs in vitro. It is worth mentioning that no obvious change of cell survival rate appeared after being incubated with 3Cys-ΔC-stabilized Au NCs at the concentration below 50 μM (Fig. 5b). Thus, 3Cys-ΔC-stabilized Au NCs could be used safely as a bioimaging agent for cells.

Fluorescent imaging in vivo is of essential importance for practical applications, and thus we further investigated the bioimaging of 3Cys-ΔC-templated Au NCs by utilizing *Caenorhabditis elegans* as an animal model (*C. elegans* is a kind of well-studied nematode with well-defined anatomy) [42, 43]. A series of worms at different cultivating times were fixed on glass slides and observed by laser confocal mircoscopy. As shown in Fig. 5c, the worms treated with two negative control samples (only *E. coli* or the complex of *E. coli* and HuFtMt mutant) showed no luminescence. Oppositely, upon treatment of the worms with the ferritin-Au NCs added *E. coli*, there existed enriching fluorescence along with the worms’ morphology from 30 to 50 h. After 70 h, the Au NCs excreted gradually, with luminescence decreasing (Additional file 1: Figure S9). For *C. elegans*, with short life-cycles, the ferritin-Au NCs composite exhibits fluorescence with high stability and comparably longer storage time. To verify the synthesized dye distribution, we detected a worm sample at the fluorescent peak by confocal microscopy. According to Fig. 5c, the ferritin-Au NCs were clearly observed in the intestines, with luminescence gathered. Differently, worms fed with only *E. coli*, complex of 3Cys-ΔC and *E. coli* showed no luminescence at all. On the other hand, worms were free living upon treatment with 3Cys-ΔC-stabilized Au NCs (Fig. 5d), indicating that the as-prepared Au NCs are safe in nature. These results demonstrate that 3Cys-ΔC-stabilized Au NCs could be absorbed by cells and *C. elegans*, while maintaining their stable fluorescence in vitro and in vivo even against complex environment. Thus, 3Cys-ΔC-stabilized Au NCs could be developed into a new class of imaging agents.

### Utilization for hybrid nanomaterials

**Fig. 6:**
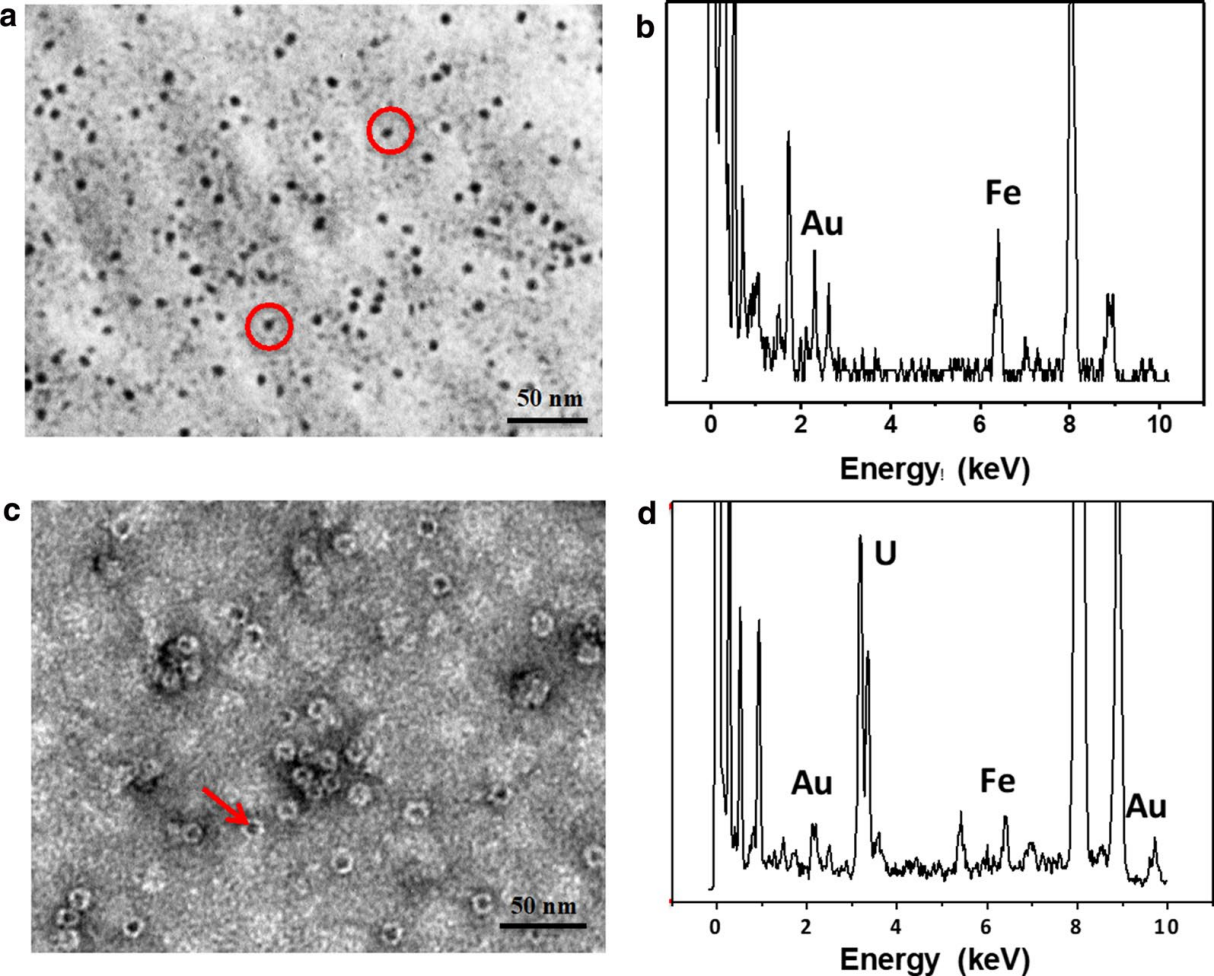
**a** TEM image and **b** EDX spectrum of iron cores formed within the cavity of the 3Cys-ΔC-templated Au NCs composite. **c** TEM image and **d** EDX spectrum of iron cores formed within the cavity of the 3Cys-ΔC-templated Au NCs composite upon negatively stained with uranyl acetate

The above observation raises an interesting question as to whether the intrinsic cavity of 3Cys-ΔC-stabilized Au NCs is still able to be used for iron mineralization after Au NCs occupy the inherent ferroxidase center located within the intrasubunit interfaces. To answer this question, we used the 3Cys-ΔC-Au NCs composite to fabricate iron cores within the inner cavity by aerobically adding 500 Fe(II) per protein shell to 3Cys-ΔC-Au NCs solutions in five successive increments of 100 Fe(II) at intervals of 30 min, followed by standing overnight based on our reported method [44]. TEM analyses revealed the formation of the electron-dense mineral cores with a diameter of 4–6 nm (Fig. 6a). Further energy-dispersive X-ray (EDX) analysis confirmed the presence of iron within the mineral cores (Fig. 6b). Simultaneously, EDX also revealed the presence of Au, which is derived from Au NCs located within the intrasubunit. After negatively stained by uranyl acetate, it was observed that iron cores are well-distributed within ferritin shell (Fig. 6c). As expected, EDX analyses revealed the co-existence of iron, gold and uranium in the sample (Fig. 6d). Thus, it appears that incorporation of Au NCs into its intrasubunit does not interfere with the formation of iron cores within the ferritin cavity. These findings raise the possibility that ferritin nanocage can be developed into a platform which are able to package two different types of inorganic nanomaterials together into a new class of hybrid nanomaterials presented in this work.

**Fig. 7:**
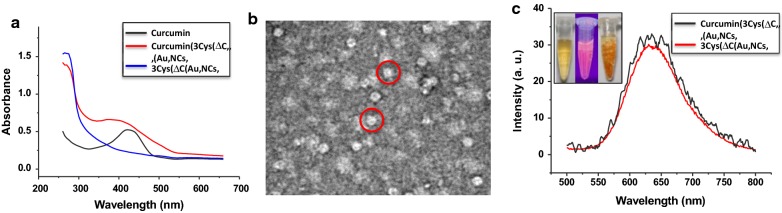
**a** UV–vis spectra of 3Cys-ΔC-stabilized Au NCs, curcumin encapsulated 3Cys-ΔC-stabilized Au NCs, and free curcumin. **b** TEM image of curcumin encapsulated within the cavity of 3Cys-ΔC-stabilized Au NCs. **c** Encapsulation of curcumin within the cavity of 3Cys-ΔC-Au NCs composite has no effect on the fluorescence spectrum of 3Cys-ΔC-stabilized Au NCs. Inset: curcumin encapsulated by 3Cys-ΔC-Au NCs under visible light and UV-light, and free curcumin (from left to right)

Except for synthesis of inorganic nanoparticles, ferritin has been also explored as a vehicle for encapsulation of drug or bioactive nutrient by taking advantage of the reversible dissociation and reassembly characteristic of apoferritin controlled by pH values as demonstrated by our and other research groups [45–47]. To shed light on effect of the formation of Au NCs within the intrasubunit interface on the ability of ferritin to trap guest molecules within the cavity, we mixed curcumin with 3Cys-ΔC-templated Au NCs at basic conditions, and then adjusted solution pH back to neutrality. It was observed that encapsulation of curcumin within ferritin cavity resulted in a color change from light yellow to deep yellow (Fig. 7c, inset). The UV–vis spectrum of the sample exhibits a new visible maximal absorption at ~ 420 nm which is characteristic of curcumin (Fig. 7a). TEM analyses revealed that black uranium-containing cores were formed in the cavity (Additional file 1: Figure S8B) with a control sample (3Cys-ΔC-Au NCs composite alone) due to uranium diffusing into the ferritin cavity via channels after being negatively stained with uranyl acetate [44]. In contrast, treatment of the ferritin-Au NCs with curcumin resulted in disappearance of the above discrete electron dense cores; instead, white nanoparticles were visualized (Fig. 7b) under the same experimental conditions, demonstrating that curcumin molecules were successfully trapped within the protein inner cavity. Interestingly, the fluorescent property of 3Cys-ΔC-templated Au NCs maintains unchanged upon curcumin encapsulation (Fig. 7c), again demonstrating that the existence of encapsulated cargo such as curcumin within ferritin shell hardly interferes with the fluorescent property of Au NCs buried within the intrasubunit, and vice versa. This is most likely derived from the fact that curcumin and Au NCs are physically separated within single ferritin molecule. To gain insights into whether the decorated protein shell with Au NCs could improve the thermal and photo stability of curcumin, we further investigated the degradation kinetics of curcumin encapsulated within the ferritin-Au NCs composite upon treatment with heating and light, respectively, with free curcumin as control. As expected, free curcumin is unstable and about 60% of free curcumin was degraded in 5 min upon thermal treatment (Additional file 1: Figure S10A). In contrast, curcumin encapsulated within ferritin cavity became much stable, and almost no degradation occurred under identical experimental conditions (Additional file 1: Figure S10B). Similarly, we found that encapsulation of curcumin by the ferritin-Au NCs composite can also pronouncedly improve its photo stability (Additional file 1: Figure S10C and S10D).

In conclusion, we built a specific compartment for fabrication of Au NCs by re-designing the intrasubunit interfaces of ferritin nanocage, resulting in the site-directed synthesis of Au NCs with protein as a biotemplate. The as-prepared Au NCs in protein shell hardly interferes with the ability of ferritin to either grow iron cores or encapsulate bioactive molecules within the protein cavity. Thus, we can incorporate two different types of nanoparticles into a single protein nanocage. These findings open a new way to prepare ‘all-in-one’ nanoparticles or nanostructures. Since the intrasubunit interface occurs in nearly all of simple proteins or multisubunit protein architectures, our engineering approach should, in principle, be applicable to these proteins. This would lead to the generation of a variety of fluorescent hybrid nanomaterials.

## Additional file

**Additional file 1: Figure S1.** Four intersubunit interfaces of ferritin: A) The *C*_4_ interface. B) The *C*_3_ interface. C) The *C*_2_ interface. D) The *C*_3_–*C*_4_ interface which is located between the *C*_3_ and *C*_4_ axes. **Figure S2.** The schematic diagram of the putative ferroxidase center buried inside four α-helix bundle of human mitochondria ferritin, which are composed of seven conserved amino acid residues. **Figure S3.** Characterization of three HuFtMt mutants ΔC, 2Cys-ΔC and 3Cys-ΔC with wt HuFtMt as control by (A) SDS PAGE, (B) Native PAGE, (C) Fluorescence spectra and (D) TEM images. Characters a, b and c correspond to ΔC, 2Cys-ΔC and 3Cys-ΔC mutants, respectively. Scale bars represent 50 nm. **Figure S4.** Fluorescence excitation spectrum of 3Cys-ΔC stabilized Au NCs with 650 nm as the maximum emission wavelength. **Figure S5.** TEM view of 3Cys-ΔC stabilized Au NCs. A) The zoom-out TEM view of 3Cys-ΔC stabilized Au NCs under basic conditions. The inset image was the distribution 3Cys-ΔC stabilized Au NCs according to their size. B) TEM view of 3Cys-ΔC stabilized Au NCs once pH was adjusted back to neutral condition. **Figure S6.** Fluorescence spectra of 3Cys-ΔC-templated Au NCs at 3 h and 8 h after adjusting solution pH to 13.5. **Figure S7.** A) The three Au ions bound at the 3-fold channels of wide-type HuFtMt. Three Cys130 are involved in coordination with the three Au ions, respectively, which are highlighted in red. B) The TEM view of Au NCs stabilized by wide-type HuFtMt. **Figure S8.** A) TEM image of 3Cys-ΔC-templated Au NCs after the solution pH was adjusted back to neutral by drop addition of 1 M acetic acid. B) EDX spectrum of the inner cavity of 3Cys-ΔC-templated Au NCs composite, where was negatively stained by uranyl acetate. **Figure S9.** Fluorescent microscopy images of C. elegans treated with 3Cys-ΔC-templated Au NCs for different times. **Figure S10.** Kinetic decay UV-vis spectra of free curcumin (A) and curcumin encapsulated 3Cys-ΔC-Au NCs (B) due to heating treatment. Kinetic decay UV-vis spectrum of free curcumin (C) and curcumin encapsulated 3Cys-ΔC-Au NCs (D) due to exposure to light. **Table S1.** X-ray diffraction data collection and processing statistics.
