# Supplementary material for: Design and site-directed compartmentalization of gold nanoclusters within the intrasubunit interfaces of ferritin nanocage
Source: J Nanobiotechnology. 2019 Jul 5;17:79. doi: 10.1186/s12951-019-0512-0 (PMC6612197; doi:10.1186/s12951-019-0512-0)

Additional Information

Design and site-directed compartmentalization of gold nanoclusters within the intrasubunit interfaces of ferritin nanocage

Jiachen Zang^1^, Bowen Zheng^1^, Xiuqing Zhang^1^, Paolo Arosio^2^, and Guanghua Zhao^1^*

***Correspondence: [gzhao@cau.edu.cn](mailto:gzhao@cau.edu.cn)

^1^Beijing Advanced Innovation Center for Food Nutrition and Human Health, College of Food Science and Nutritional Engineering, China Agricultural University, Beijing Key Laboratory of Functional Food from Plant Resources, Beijing, 100083, China

^2^Department of Molecular and Translational Medicine, University of Brescia, Viale Europa 11, Brescia, 25123, Italy

The file includes

1. Figure S1-S10

2. Table S1

**
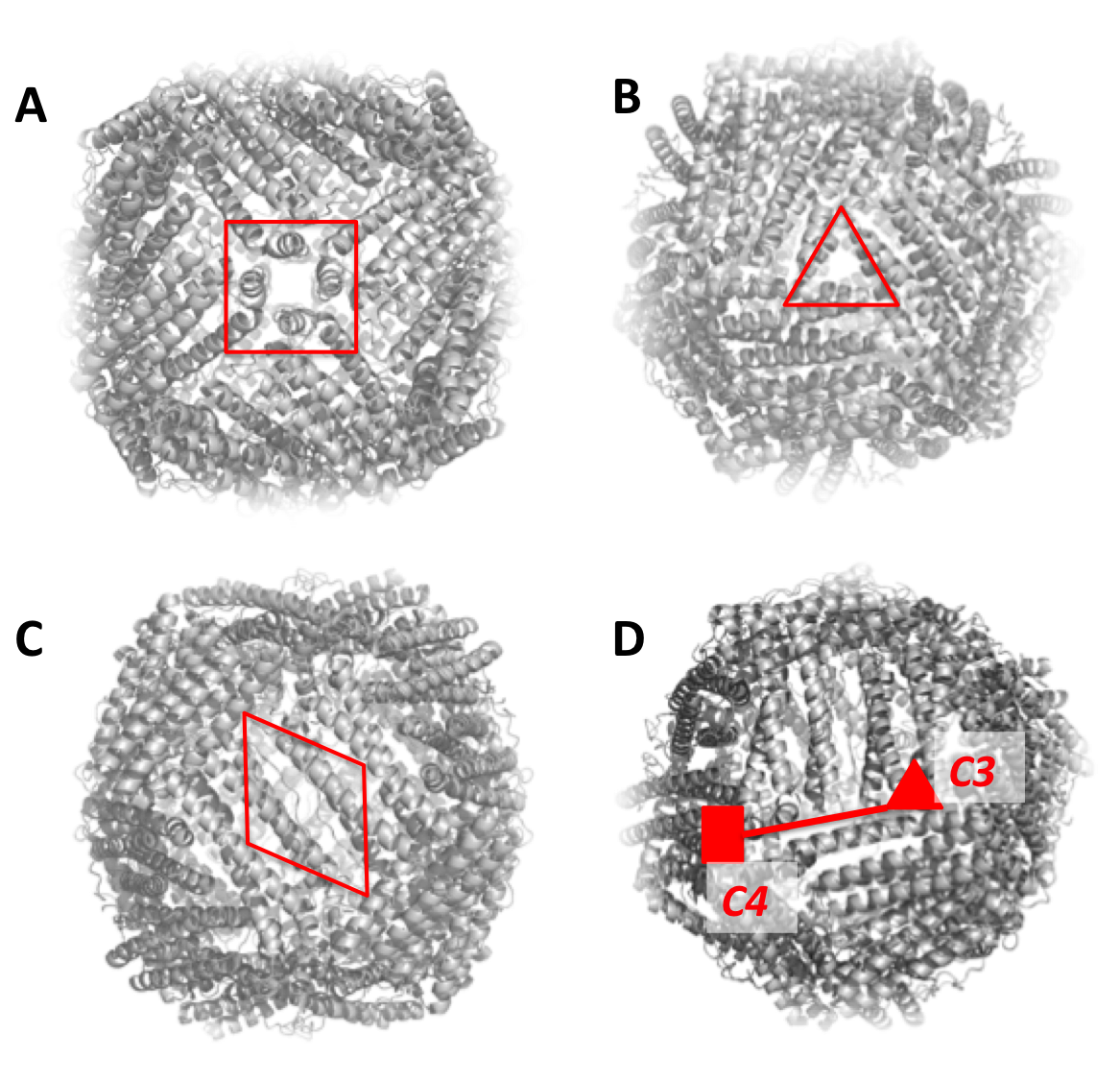
**

**Figure S1.** Four intersubunit interfaces of ferritin: A) The *C_4_* interface. B) The *C_3_* interface. C) The *C_2_* interface. D) The *C_3_-C_4_* interface which is located between the *C_3_* and *C_4_* axes.

**
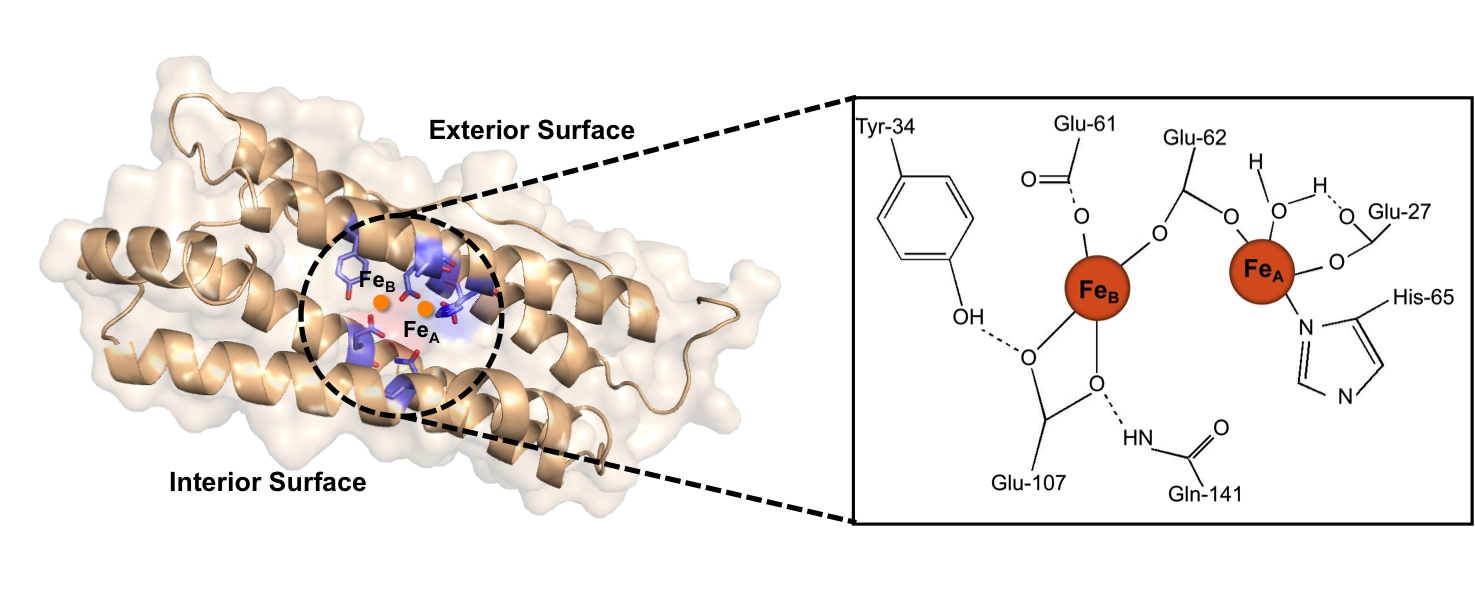
**

**Figure S2.** The schematic diagram of the putative ferroxidase center buried inside four α-helix bundle of human mitochondria ferritin, which are composed of seven conserved amino acid residues .

**
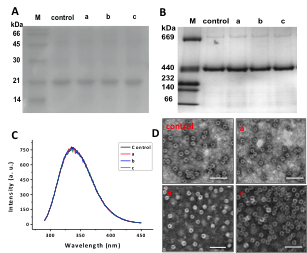
**

**Figure S3.** Characterization of three HuFtMt mutants ΔC, 2Cys-ΔC and 3Cys-ΔC with wt HuFtMt as control by (A) SDS PAGE, (B) Native PAGE, (C) Fluorescence spectra and (D) TEM images. Characters a, b and c correspond to ΔC, 2Cys-ΔC and 3Cys-ΔC mutants, respectively. Scale bars represent 50 nm.


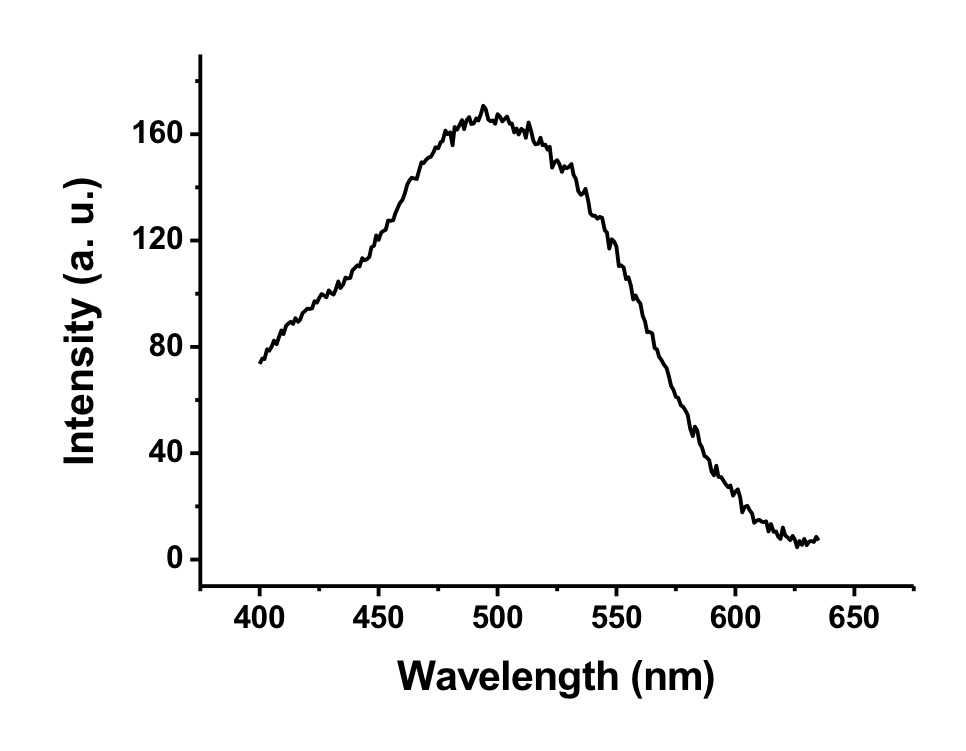


**Figure S4.** Fluorescence excitation spectrum of 3Cys-ΔC stabilized Au NCs with 650 nm as the maximum emission wavelength.


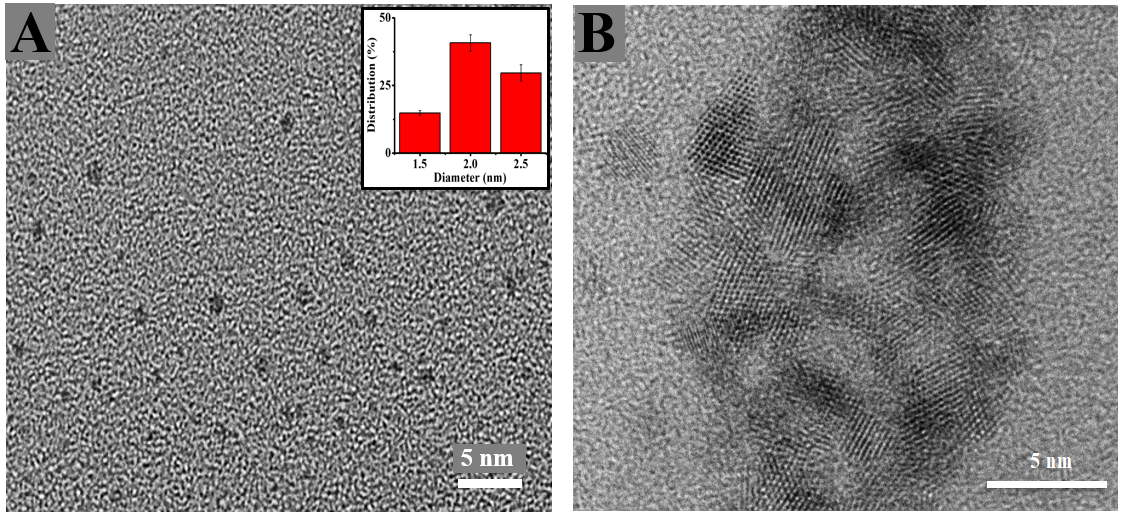


**Figure S5.** TEM view of 3Cys-ΔC stabilized Au NCs. A) The zoom-out TEM view of 3Cys-ΔC stabilized Au NCs under basic conditions. The inset image was the distribution 3Cys-ΔC stabilized Au NCs according to their size. B) TEM view of 3Cys-ΔC stabilized Au NCs once pH was adjusted back to neutral condition.


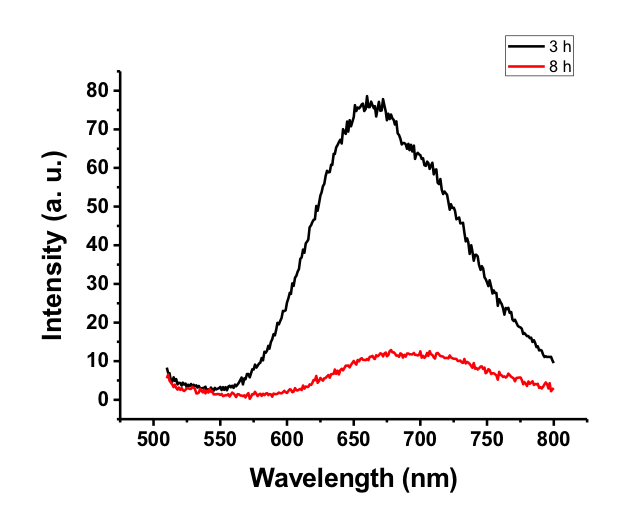


**Figure S6.** Fluorescence spectra of 3Cys-ΔC-templated Au NCs at 3 h and 8 h after adjusting solution pH to 13.5.


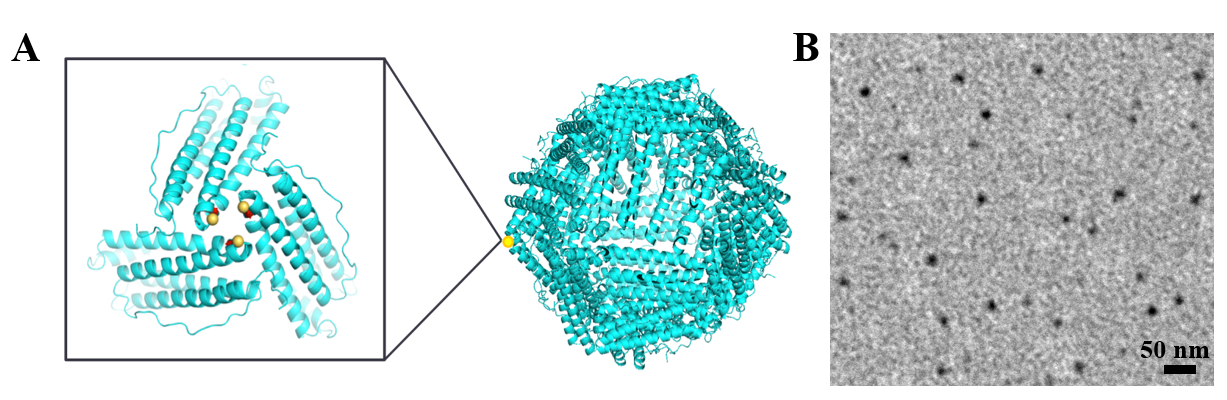


**Figure S7.** A) The three Au ions bound at the 3-fold channels of wide-type HuFtMt. Three Cys130 are involved in coordination with the three Au ions, respectively, which are highlighted in red. B) The TEM view of Au NCs stabilized by wide-type HuFtMt.


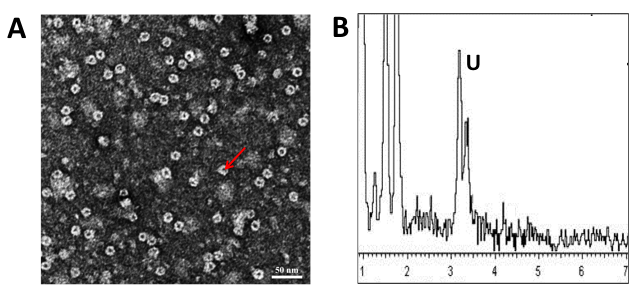


**Figure S8.** A) TEM image of 3Cys-ΔC-templated Au NCs after the solution pH was adjusted back to neutral by drop addition of 1 M acetic acid. B) EDX spectrum of the inner cavity of 3Cys-ΔC-templated Au NCs composite, where was negatively stained by uranyl acetate.


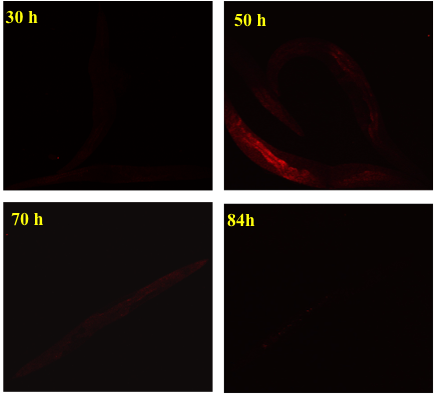


**Figure S9.** Fluorescent microscopy images of C. elegans treated with 3Cys-ΔC-templated Au NCs for different times.


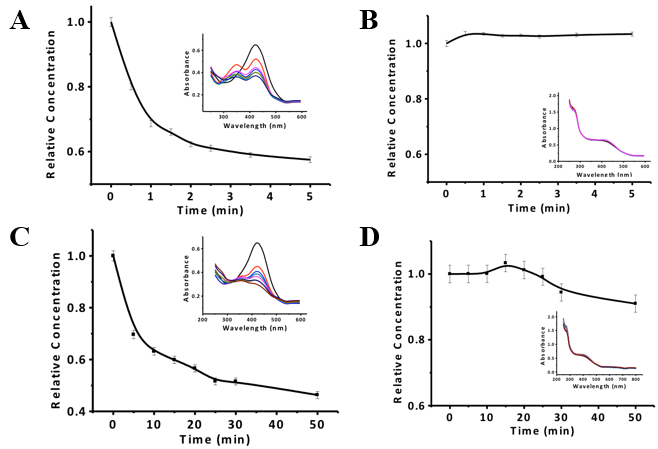


**Figure S10.** Kinetic decay UV-vis spectra of free curcumin (A) and curcumin encapsulated 3Cys-ΔC-Au NCs (B) due to heating treatment. Kinetic decay UV-vis spectrum of free curcumin (C) and curcumin encapsulated 3Cys-ΔC-Au NCs (D) due to exposure to light.

**Table S1.** X-ray diffraction data collection and processing statistics.


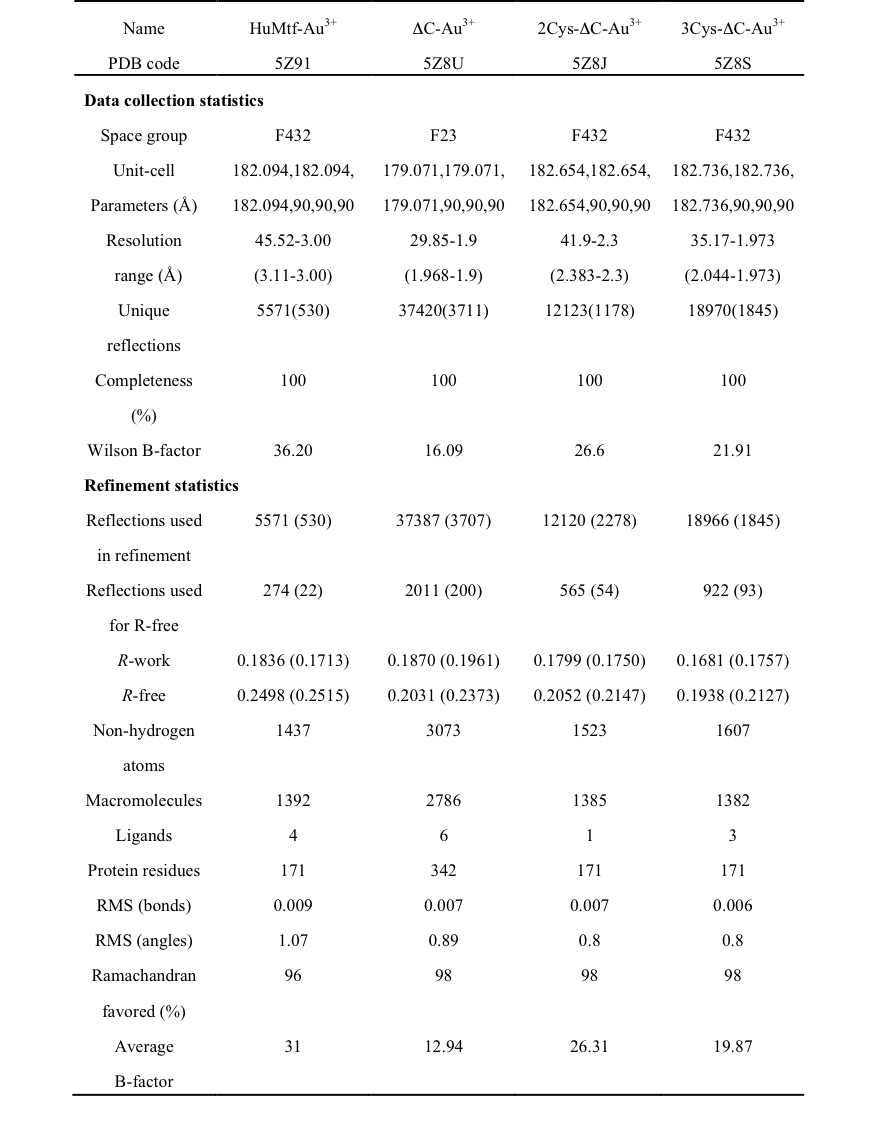

Supplement: Supplementary file 1 — Additional file 1: Figure S1. Four intersubunit interfaces of ferritin: A) The C4 interface. B) The C3 interface. C) The C2 interface. D) The C3–C4 interface which is located between the C3 and C4 axes. Figure S2. The schematic diagram of the putative ferroxidase center buried inside four α-helix bundle of human mitochondria ferritin, which are composed of seven conserved amino acid residues. Figure S3. Characterization of three HuFtMt mutants ΔC, 2Cys-ΔC and 3Cys-ΔC with wt HuFtMt as control by (A) SDS PAGE, (B) Native PAGE, (C) Fluorescence spectra and (D) TEM images. Characters a, b and c correspond to ΔC, 2Cys-ΔC and 3Cys-ΔC mutants, respectively. Scale bars represent 50 nm. Figure S4. Fluorescence excitation spectrum of 3Cys-ΔC stabilized Au NCs with 650 nm as the maximum emission wavelength. Figure S5. TEM view of 3Cys-ΔC stabilized Au NCs. A) The zoom-out TEM view of 3Cys-ΔC stabilized Au NCs under basic conditions. The inset image was the distribution 3Cys-ΔC stabilized Au NCs according to their size. B) TEM view of 3Cys-ΔC stabilized Au NCs once pH was adjusted back to neutral condition. Figure S6. Fluorescence spectra of 3Cys-ΔC-templated Au NCs at 3 h and 8 h after adjusting solution pH to 13.5. Figure S7. A) The three Au ions bound at the 3-fold channels of wide-type HuFtMt. Three Cys130 are involved in coordination with the three Au ions, respectively, which are highlighted in red. B) The TEM view of Au NCs stabilized by wide-type HuFtMt. Figure S8. A) TEM image of 3Cys-ΔC-templated Au NCs after the solution pH was adjusted back to neutral by drop addition of 1 M acetic acid. B) EDX spectrum of the inner cavity of 3Cys-ΔC-templated Au NCs composite, where was negatively stained by uranyl acetate. Figure S9. Fluorescent microscopy images of C. elegans treated with 3Cys-ΔC-templated Au NCs for different times. Figure S10. Kinetic decay UV-vis spectra of free curcumin (A) and curcumin encapsulated 3Cys-ΔC-Au NCs (B) due to heating treatment. Kinetic decay [file 12951_2019_512_MOESM1_ESM.doc]
